# Inflammation, sex, blood pressure changes and hypertension in midlife: the Hordaland Health Study

**DOI:** 10.1038/s41371-022-00772-z

**Published:** 2022-11-18

**Authors:** Ester Kringeland, Eva Gerdts, Arve Ulvik, Grethe S. Tell, Jannicke Igland, Teresa R. Haugsgjerd, Per Magne Ueland, Helga Midtbø

**Affiliations:** 1 Department of Clinical Science, University of Bergen, Bergen, Norway; 2 BEVITAL, Bergen, Norway; 3 Department of Global Public Health and Primary Care, University of Bergen, Bergen, Norway; 4 Department of Heart Disease, Haukeland University Hospital, Bergen, Norway

**Keywords:** Risk factors, Diseases

## Abstract

Our aim was to test sex-specific associations of circulating markers of inflammation with blood pressure (BP) and incident hypertension in midlife. Participants in the Hordaland Health study (*n* = 3280, 56% women, mean age 48 years) were examined at baseline and followed for 6 years. Circulating levels of inflammatory markers including high-sensitive C-reactive protein (hs-CRP), neopterin, and pyridoxic acid ratio (PAr) index were measured at follow-up. The associations with systolic/diastolic BP and incident hypertension were tested in sex-specific linear- or logistic-regression analyses adjusted for body mass index, serum triglycerides, creatinine, physical activity, smoking and diabetes. At follow-up, women had lower mean BP than men (124/72 vs. 130/78 mmHg, *p* < 0.001). Higher hs-CRP was significantly associated with greater systolic and diastolic BP (standardized *β* = 0.07 and *β* = 0.09, both *p* < 0.01) in women, but not in men. Higher neopterin was associated with higher diastolic BP in women and higher PAr index was associated with higher diastolic BP in women and higher systolic and diastolic BP in men (all *p* < 0.01). Compared to hs-CRP < 1 mg/l, higher levels of hs-CRP 1–<3 mg/l and hs-CRP ≥ 3 mg/l were associated with new-onset hypertension only in women (odds ratio (OR) 1.74, 95% CI 1.20–2.53 and OR 1.87, 95% CI 1.20–2.90). Sex-interactions were found for hs-CRP and neopterin in models on incident hypertension and diastolic BP, respectively (both *p* < 0.05). Higher levels of circulating markers of inflammation were associated with higher BP and incident hypertension in a sex-specific manner. Our results suggest a sex-specific interaction between cardiovascular inflammation and BP in midlife.

## Introduction

Elevated blood pressure (BP) is the leading risk factor for cardiovascular disease and mortality [1]. Sex differences in BP development over the life course have been identified, where women have a steeper increase in BP with aging, starting already in the third decade of life [2]. Further, we recently reported that the risk for acute coronary syndromes

increased at a lower BP level in women than in men participating in the community-based Hordaland Health Study [3]. The underlying causes for these sex-differences in BP development have not been established; however, evidence from experimental studies indicates that sex-specific activation of the immune system may contribute [4, 5]. In particular, vascular activation of T cells in response to stimuli like angiotensin 2 and high salt intake seems to be central for BP development in animal studies [4]. These activated T cells may modulate the BP response through production of pro-inflammatory cytokines such as interferon gamma (IFN-γ) that stimulates production of neopterin by macrophages, and metabolism of the essential amino acid tryptophan through the kynurenine pathway [6, 7]. The degree of T cell activation may be assessed through measurement of neopterin and the kynurenine-tryptophan ratio (KTR) [8].

Previous studies in community-based cohorts have documented that elevated circulating levels of high-sensitive C-reactive protein (hs-CRP), neopterin, KTR and the pyridoxic acid ratio (PAr index), the latter reflecting altered vitamin B6 catabolism during inflammation, are associated with high BP [9–14]. Furthermore, elevated circulating levels of these inflammatory markers have been reported to be associated with risk of cardiovascular disease [11, 12, 14, 15, 16]. However, there is a paucity of clinical information on the sex-specific associations between these prognostic inflammatory markers and BP development. In the present analysis, we explored the sex-specific associations of prognostically validated circulating markers of inflammation with systolic and diastolic BP and new onset hypertension over the past 6 years in middle-aged women and men participating in the Hordaland Health Study [17].

## Methods

### Study population

The community-based Hordaland Health Study was initiated in Hordaland County in Western Norway as a collaboration between the National Health Screening Service, the University of Bergen and local health services in 1992 (https://husk-en.w.uib.no/). Eligible subjects were identified from the National Population Registry based on year of birth and site of residence. The cohort used in the present analysis includes 3700 women and men born in 1950–1951 participating in the 1st and 2nd Hordaland Health Survey in 1992–1993 and 1997–1999, respectively [17]. Participation rate was above 70% in both surveys. We excluded participants with missing data on BP (*n* = 6), circulating markers of inflammation (*n* = 264) body mass index (BMI) (*n* = 4), serum creatinine (*n* = 1) and physical activity (*n* = 164), leaving 1829 women and 1451 men eligible for the present study. The study was performed according to the declaration of Helsinki, and the study protocol was approved by the Regional Committee for Medical and Health Research Ethics (2017/294). All participants provided written informed consent.

### Blood pressure measurement

Study participants were examined following standardized protocols in 1992–1993 and 1997–1999. Attended brachial BP was measured in seated position after a minimum of 2 min rest with calibrated sphygmomanometers (Dinamap 845 XT or Dinamap 8100, Criticon, Tampa, FL, USA). BP was measured three times by trained health care workers, and the average of the two last measurements was used for analyses. Hypertension was defined as BP ≥ 130/80 mmHg or self-reported use of antihypertensive drugs [18].

### Other cardiovascular disease risk factors

Information about use of antihypertensive medication in 1992–1993 (baseline) was collected in self-reported questionnaires. Likewise, information about physical activity, smoking, medical history and use of medication in 1997–1999 (follow-up) was collected in self-reported questionnaires. Physical activity was categorized as none, <1 h, 1–2 h or ≥3 h per week of vigorous physical activity resulting in sweating or shortness of breath. Smoking was defined as self-reported daily smoking or serum cotinine ≥85 nmol/l [19]. Diabetes was defined as self-reported diabetes, use of antidiabetic medication, a serum glucose level ≥11.1 mmol/l in participants who had eaten during the last 8 h and/or a serum glucose level ≥7.0 mmol/l in participants who had been fasting for at least 8 h. Weight was measured with light clothing to the nearest half-kilogram, and height was measured without shoes to the nearest centimeter. Body mass index (BMI) was calculated as weight in kg/height in meters^2^ and obesity was defined as BMI ≥ 30 kg/m^2^. Serum triglycerides were measured in non-fasting blood samples.

### Biochemical analyses

Non-fasting blood samples were collected at follow-up, kept on ice before centrifugation (for less than 3 h), and stored at −80 °C before analysis.

Plasma concentrations of tryptophan, kynurenine, neopterin, 4-pyridoxic acid, pyridoxal 5’-phosphate, pyridoxal, cotinine, and creatinine were quantified by LC-tandem MS at Bevital, Bergen, Norway (www.bevital.no) [20]. Plasma hs-CRP was measured with an immuno-Matrix-Assisted Laser Desorption/Ionization–based assay [21]. KTR was calculated by dividing the plasma concentration of kynurenine (nmol/l) by the concentration of tryptophan (mmol/l), and the PAr index was defined as the ratio plasma 4-pyridoxic acid: (pyridoxal + pyridoxal-5’-phosphate) [10].

### Statistical analyses

Statistical analyses were done using STATA, version 17 (Stata Corp LP, College Station, TX, USA). Continuous variables are expressed as means and standard deviations (SD) or medians and interquartile ranges (IQR) for variables (serum triglycerides, hs-CRP, neopterin, KTR and PAr) not normally distributed. Categorical variables are presented as numbers and percentages. Comparisons between groups were done using the Student’s *t*-test or the Chi-square test. For non-normally distributed variables comparisons of medians between groups were done using quantile regression. Cross-sectional associations between inflammatory markers and systolic and diastolic BP measured at follow-up were tested in univariable and multivariable linear regression analyses. Likewise, associations between inflammatory markers measured at follow-up with changes in systolic and diastolic BP during the past 6 years were tested by linear regression analyses. Results are reported as standardized *β*-coefficients and *p* values. For the analysis of new onset hypertension at follow-up, participants with hypertension at baseline were excluded (*n* = 1599). Associations between inflammatory markers and new onset hypertension at follow-up were tested by logistic regression analyses. Results are reported as odds ratios (OR), 95% confidence intervals (CI) and *p* values. The inflammatory markers were all non-normally distributed and log-transformed before inclusion in linear or logistic regression analyses. Separate analyses were performed for women and men. To test for interactions with sex in the associations between circulating markers of inflammation and blood pressure/hypertension, we compared a model with and without an interaction term, using the likelihood-ratio test. Multivariable model 1 was adjusted for BMI. Model 2 was adjusted for BMI, serum creatinine, physical activity, daily smoking, diabetes, and non-fasting serum triglycerides. In addition, multivariable models on changes in systolic and diastolic BP were adjusted for systolic or diastolic BP measured at baseline, respectively. We performed sensitivity analyses after exclusion of participants taking antihypertensive medication at baseline and/or follow up (*n* = 169) and of participants with prior myocardial infarction and stroke (*n* = 32).

## Results

### Characteristics of the study population in the Hordaland Health Study

At follow-up, women had lower systolic and diastolic BP and lower prevalence of hypertension compared to men (all *p* < 0.001) (Table 1). In participants with hypertension, 13% of women and 9% of men were taking antihypertensive medication (*p* = 0.03). Women had lower hs-CRP and higher neopterin and PAr levels than men (all *p* < 0.05), while KTR did not differ according to sex (*p* < 0.05) (Table 1). Women also had lower BMI and serum creatinine (both *p* < 0.001) (Table 1).

**Table 1:** Characteristics of the study population at follow-up in 1997–1999: the Hordaland Health Study.

	Women *n* = 1829	Men *n* = 1451	*p* for sex difference
Age, years	48 ± 1	48 ± 1	0.36
Systolic BP, mmHg	124 ± 16	130 ± 14	<0.01
Diastolic BP, mmHg	72 ± 11	78 ± 10	<0.01
Δ systolic BP during 6 years follow-up	1.8 ± 12	−1.3 ± 11	<0.01
Δ diastolic BP during 6 years follow-up	−3.8 ± 8.4	−1.7 ± 8.6	<0.01
Hypertension, *n* (%)	670 (37)	836 (58)	<0.01
Patients with treated hypertension, *n* (%)	85 (13)	77 (9)	0.03
Body mass index, kg/m^2^	24.8 ± 4.0	26.1 ± 3.1	<0.01
Obesity, *n* (%)	195 (10)	184 (11)	0.13
Creatinine, mmol/l	82 ± 9	95 ± 11	<0.01
Serum triglycerides, median (IQR), mmol/l	1.2 (0.9–1.7)	1.8 (1.2–2.5)	<0.01
Smoking, *n* (%)	673 (37)	552 (38)	0.46
Diabetes, *n* (%)	10 (0.55)	27 (1.9)	<0.01
Physical activity, *n* (%)			<0.01
None	554 (30)	336 (23)	
<1 h/week	480 (26)	443 (31)	
1–2 h/week	580 (32)	425 (29)	
≥3 h/week	215 (12)	247 (17)	
Myocardial infarction, *n* (%)	1 (0.06)	16 (1.11)	<0.01
Stroke, *n* (%)	10 (0.55)	5 (0.34)	0.56
hs-CRP, median (IQR) mg/l	1.01 (0.43–2.56)	1.15 (0.53–2.62)	0.02
Neopterin, median (IQR) nmol/l	7.07 (6.02–8.36)	6.69 (5.72–7.86)	<0.01
Kynurenine, median (IQR) µmol/l	1.39 (1.23–1.57)	1.53 (1.36–1.71)	<0.01
Tryptophan, median (IQR) µmol/l	68.2 (60.8–76.6)	75.8 (67.5–84.1)	<0.01
KTR, median (IQR) nmol/µmol	20.1 (18.0–22.6)	20.3 (18.0–22.5)	0.38
PAr, median (IQR)	0.36 (0.28–0.47)	0.31 (0.25–0.41)	<0.01

Values are given as means (SD) unless otherwise indicated.

*BP* blood pressure, *Hypertension* BP ≥ 130/80 mmHg or use of antihypertensive medication, *hs-CRP* high-sensitive C-reactive protein, *KTR* kynurenine:tryptophan ratio, *PAr index* ratio 4-pyridoxic acid/(pyridoxal 5’-phosphate + pyridoxal), *SD* standard deviation, *IQR* interquartile range.

### Association of BP with circulating markers of inflammation in women and men

#### Women

**Fig. 1:**
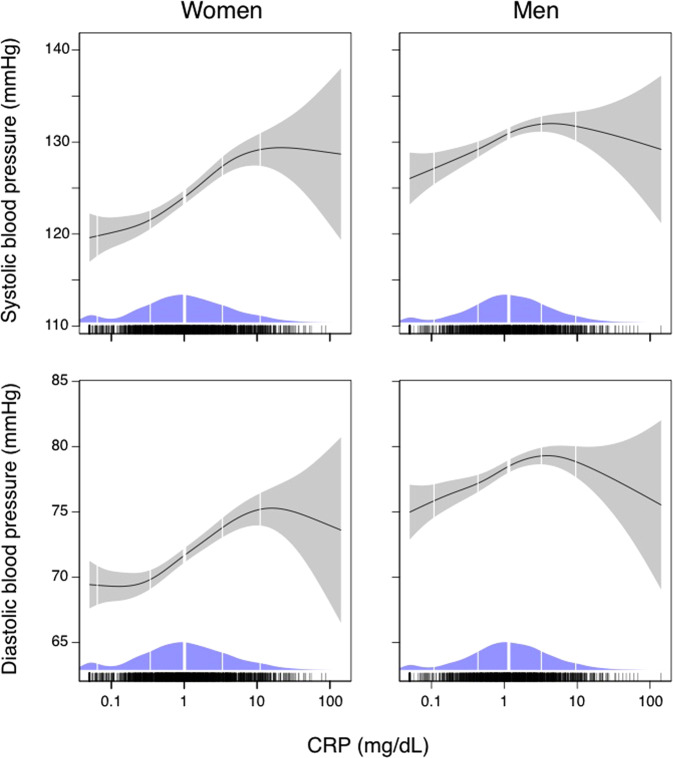
GAM plots demonstrating univariable associations of hs-CRP with systolic and diastolic blood pressure in 46–49 years old women and men in the Hordaland Health Study. The solid lines indicate blood pressure and the shaded areas 95% confidence intervals. Density plots indicate distributions, and the vertical white lines indicate the 5th, 20th, 50th, 80th and 95th percentiles of hs-CRP. CRP high-sensitive C-reactive protein.

**Fig. 2:**
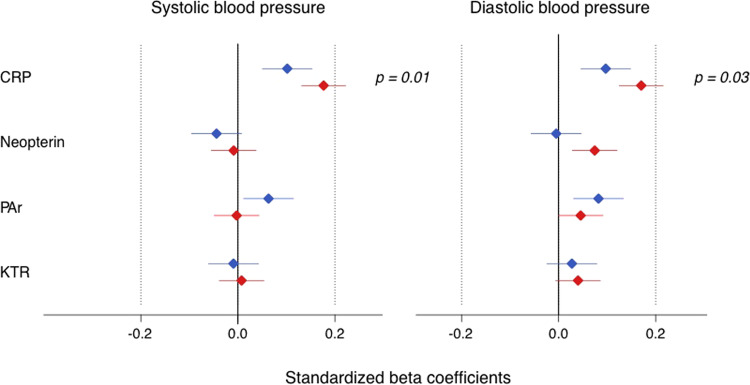
Associations of four circulating markers of inflammation (hs-CRP, neopterin, PAr index and KTR) with systolic and diastolic blood pressure in 46–49 years old women and men in the Hordaland Health Study. Red (women) and blue (men) diamonds indicate standardized beta coefficients and lines 95% confidence intervals from univariable linear regression analysis, respectively. *p* *p* for interaction between biomarker and sex, CRP high-sensitive C-reactive protein, PAr the ratio plasma 4-pyridoxic acid: (pyridoxal + pyridoxal-5’-phosphate), KTR kynurenine:tryptophan ratio.

Univariable results are presented in Table 2 and Figs. 1 and 2. Higher hs-CRP was significantly associated with higher systolic and diastolic BP after adjustment for creatinine, physical activity, daily smoking, diabetes, and non-fasting serum triglycerides (Table 2, model 2) (*β* = 0.07 for systolic, and *β* = 0.09 for diastolic BP, respectively, both *p* < 0.01). Higher plasma neopterin and higher PAr index was associated with higher diastolic BP (*β* = 0.09, and *β* = 0.05, both *p* < 0.01). No significant associations between KTR and systolic (*β* = −0.03) or diastolic (*β* = 0.02) BP was found (both *p* > 0.05) (Table 2).

**Table 2:** Association of systolic and diastolic BP with levels of circulating markers of inflammation in 46–49 years old women and men: the Hordaland Health study.

	Women						Men						Interaction biomarker and sex		
Variable	Univariable		Model 1		Model 2		Univariable		Model 1		Model 2		Univariable	Model 1	Model 2
	*β*	*p*	*β*	*p*	*β*	*p*	*β*	*p*	*β*	*p*	*β*	*p*	*p*	*p*	*p*
Systolic BP															
hs-CRP	**0.172**	**<0.01**	**0.081**	**<0.01**	**0.072**	**<0.01**	**0.102**	**<0.01**	0.036	0.17	0.038	0.16	**0.03**	0.37	0.50
Neopterin	−0.001	0.98	0.001	0.97	−0.004	0.85	−0.039	0.13	−0.037	0.14	−0.038	0.15	0.30	0.29	0.38
KTR	0.013	0.58	−0.032	0.16	−0.031	0.18	−0.009	0.72	−0.025	0.32	−0.027	0.31	0.53	0.67	0.79
PAR index	−0.002	0.95	0.004	0.87	0.007	0.75	**0.065**	**0.01**	**0.065**	**<0.01**	**0.066**	**<0.01**	0.07	0.07	0.08
Diastolic BP															
hs-CRP	**0.165**	**<0.01**	**0.101**	**<0.01**	**0.087**	**<0.01**	**0.094**	**<0.01**	0.04	0.13	0.042	0.13	**0.04**	0.14	0.20
Neopterin	**0.084**	**<0.01**	**0.085**	**<0.01**	**0.087**	**<0.01**	−0.004	0.87	−0.002	0.93	0.008	0.76	**0.01**	**0.02**	**0.01**
KTR	**0.049**	**0.04**	0.015	0.51	0.021	0.38	0.023	0.38	0.010	0.70	0.019	0.49	0.41	0.99	0.97
PAR index	0.045	0.06	**0.049**	**0.03**	**0.049**	**0.03**	**0.079**	**<0.01**	**0.084**	**<0.01**	**0.089**	**<0.01**	0.34	0.35	0.39

Model 1 is adjusted for body mass index. Model 2 is in addition adjusted for serum creatinine, physical activity, daily smoking, diabetes, and non-fasting serum triglycerides.

*BP* blood pressure, *hs-CRP* high-sensitive C-reactive protein, *PAr index* ratio: 4-pyridoxic acid/(pyridoxal 5’-phosphate + pyridoxal), *KTR* kynurenin:tryptophan ratio.

Significant associations are printed in bold.

#### Men

Univariable results are presented in Table 2 and Figs. 1 and 2. In adjusted analyses, higher hs-CRP was neither associated with higher systolic nor diastolic BP (both *β* = 0.04, *p* > 0.05) (Table 2, model 2). A higher plasma PAr index was associated with higher systolic (*β* = 0.07) and diastolic (*β* = 0.09) BP (both *p* < 0.01) (Table 2). There were no significant associations between plasma neopterin with systolic (*β* = −0.04) or diastolic (*β* = 0.008) BP (both *p* > 0.05) (Table 2, model 2). Further, there were no associations between KTR with systolic (*β* = −0.03) or diastolic (*β* = 0.02) BP in men (both *p* > 0.05) (Table 2, model 2).

Significant interactions between plasma hs-CRP and sex were found in univariable analyses for systolic and diastolic BP (both *p* < 0.05). A significant sex-interaction with neopterin was found in the model on diastolic BP (*p* = 0.01) (Table 2), indicating that neopterin has a significantly stronger association with diastolic BP in women compared to men.

### Association of BP changes over the prior 6 years with circulating markers of inflammation in women and men

Over 6 years follow-up, women had an increase in systolic BP and a decrease in diastolic BP, while men had decreases in both systolic and diastolic BP (both *p* < 0.01 for sex-difference) (Table 1).

#### Women

Univariable results are presented in Table 3. In adjusted analysis, higher hs-CRP at follow-up was associated with larger increases in systolic (*β* = 0.07) and diastolic (*β* = 0.08) BP over the prior 6 years (both *p* < 0.01) (Table 3, model 2). Higher plasma neopterin was associated with a larger increase in diastolic BP (*β* = 0.07) over the prior 6 years (*p* < 0.01) (Table 3). Neither the PAr index nor KTR ratio were associated with changes in systolic or diastolic BP over the prior 6 years (all *p* > 0.05).

**Table 3:** Association of changes in systolic and diastolic BP over the prior 6 years with levels of circulating markers of inflammation in 46–49 years old women and men: the Hordaland Health Study.

	Women						Men						Interaction biomarker and sex		
Variable	Univariable		Model 1		Model 2		Univariable		Model 1		Model 2		Univariable	Model 1	Model 2
	*β*	*p*	*β*	*p*	*β*	*p*	*β*	*p*	*β*	*p*	*β*	*p*	*p*	*p*	*p*
Δ systolic BP															
hs-CRP	**0.102**	**<0.01**	**0.067**	**<0.01**	**0.072**	**<0.01**	0.016	0.54	−0.005	0.87	0.015	0.66	**0.01**	**0.047**	0.10
Neopterin	0.023	0.32	0.024	0.30	0.018	0.40	−0.012	0.64	−0.011	0.66	0.008	0.75	0.32	0.32	0.27
KTR	−0.006	0.81	−0.026	0.27	−0.037	0.11	−0.039	0.14	−0.044	0.093	−0.043	0.09	0.38	0.64	0.73
PAR index	0.012	0.60	0.015	0.53	0.011	0.63	0.024	0.36	0.026	0.33	0.047	0.05	0.76	0.76	0.37
Δ diastolic BP															
hs-CRP	**0.054**	**0.02**	**0.058**	**0.02**	**0.079**	**<0.01**	−0.023	0.38	−0.029	0.28	0.006	0.80	**0.03**	**0.03**	0.09
Neopterin	**0.065**	**<0.01**	**0.065**	**<0.01**	**0.074**	**<0.01**	0.026	0.33	0.026	0.32	0.022	0.37	0.02	0.30	0.08
KTR	0.006	0.81	0.004	0.88	0.005	0.83	0.001	0.98	−0.0004	0.99	0.008	0.74	0.89	0.93	0.81
PAR index	0.014	0.54	0.014	0.54	0.027	0.22	0.039	0.14	0.039	0.14	**0.072**	**<0.01**	0.45	0.45	0.31

Model 1 is adjusted for body mass index. Model 2 is adjusted for body mass index, serum creatinine, physical activity, daily smoking, diabetes, and non-fasting serum triglycerides. In addition model 2 is adjusted systolic or diastolic BP at baseline in the models on change in systolic and diastolic BP, respectively.

*β* standardized *B* coefficient, *BP* blood pressure, *hs-CRP* high-sensitive C-reactive protein, *PAr index* ratio: 4-pyridoxic acid/(pyridoxal 5’-phosphate + pyridoxal), *KTR* kynurenin:tryptophan ratio.

Significant associations are printed in bold.

#### Men

Univariable results are presented in Table 3. In contrast to findings in women, hs-CRP was not associated with increases in systolic (*β* = 0.02) or diastolic (*β* = 0.006) BP over the prior 6 years in men (*p* > 0.05) (Table 3, model 2). Neopterin was also not associated with prior increases in systolic (*β* = 0.008) or diastolic (*β* = 0.02) BP. Higher PAr index was associated with a larger increase in diastolic BP (*β* = 0.07) (*p* < 0.05) over the prior 6 years, while KTR was not associated with change in systolic or diastolic BP (*p* > 0.05) (Table 3).

A significant sex-interaction with hs-CRP was found in univariable analyses for changes in systolic and diastolic BP (both *p* < 0.05).

### Associations of new onset hypertension with circulating markers of inflammation in women and men

Among 1155 women and 526 men who were normotensive and free from antihypertensive medication at baseline, 17% of women and 30% of men developed new-onset hypertension during 6 years follow-up (*p* < 0.01). Only 7% of women and 5% of men with new onset hypertension at follow-up were taking antihypertensive medication (*p* = 0.37). Among participants not taking antihypertensive medication, 12% of women and 23% of men had stage 1 hypertension (BP 130–139/80–89 mmHg) (*p* < 0.01), and 5% of women and 6% of men had stage 2 hypertension (BP ≥ 140/90 mmHg) (*p* = 0.32).

Among women, new-onset hypertension was associated with higher plasma hs-CRP (OR 1.18 95% CI 1.04–1.34) (Model 2, Table 4). Both women with hs-CRP 1–<3 mg/l (OR 1.74, 95% CI 1.20–2.53) and hs-CRP ≥ 3 mg/l (OR 1.87, 95% CI 1.20–2.90) had an increased risk of new-onset hypertension compared to women having hs-CRP < 1 mg/l (Model 2, Table 4). Neopterin, KTR or the PAr index were not associated with new onset hypertension in women (all *p* > 0.05). Among men, neither hs-CRP, neopterin, KTR nor the PAr index were associated with new onset hypertension (all *p* > 0.05) (Table 4). A significant interaction between hs-CRP and sex was found in models on incident hypertension, confirming a sex-specific association between hs-CRP and hypertension (all *p* < 0.05).

**Table 4:** Association of new onset hypertension with levels of circulating markers of inflammation in 46–49 years old women and men: the Hordaland Health Study.

	Women *n* = 1155				Men *n* = 526				Interaction biomarker and sex	
Variable	Univariable		Model 2		Univariable		Model 2		Univariable	Model 2
	OR 95% CI	*p*	OR 95% CI	*p*	OR 95% CI	*p*	OR 95% CI	*p*	*p*	*p*
hs-CRP^a^	**1.34 (1.20–1.50)**	**<0.01**	**1.18 (1.04–1.34)**	**<0.01**	1.04 (0.91–1.18)	<0.61	0.97 (0.84–1.12)	0.71	**<0.01**	**0.046**
hs-CRP										
<1 mg/l	**Ref**		**Ref**		Ref		Ref		**<0.01**	**0.04**
1–<3 mg/l	**2.02 (1.41–2.90)**	**<0.01**	**1.74 (1.20–2.53)**	**<0.01**	1.40 (0.93–2.14)	0.11	1.22 (0.79–1.90)	0.37		
≥3 mg/l	**2.91 (1.98–4.29)**	**<0.01**	**1.87 (1.20–2.90)**	**<0.01**	1.08 (0.65–1.79)	0.78	0.87 (0.49–1.53)	0.62		
Neopterin^a^	1.06 (0.61–1.84)	0.83	1.02 (0.58–1.81)	0.97	0.90 (0.45–1.82)	0.77	0.83 (0.39–1.74)	0.85	0.71	0.69
KTR^a^	1.92 (0.85–4.32)	0.11	1.11 (0.46–2.67)	0.82	0.90 (0.32–2.49)	0.83	0.71 (0.24–2.09)	0.53	0.25	0.59
PAr index^a^	1.34 (0.93–1.93)	0.11	1.31 (0.90–1.90)	0.16	0.99 (0.60–1.63)	0.97	1.00 (0.59–1.68)	0.99	0.34	0.43

Model 2 is adjusted for body mass index, creatinine, physical activity, daily smoking, diabetes, and non-fasting serum triglycerides. For this analysis participants with hypertension at baseline were excluded.*Hypertension* BP ≥ 130/80 mmHg or use of antihypertensive medication, *OR* odds ratio, *CI* confidence interval, *hs-CRP* high-sensitive C-reactive protein, *PAr index* ratio: 4-pyridoxic acid/(pyridoxal 5’-phosphate + pyridoxal), *KTR* kynurenin:tryptophan ratio.

^a^OR per 1 unit increase on log-transformed scale for each inflammation marker.

Significant associations are printed in bold.

### Associations of antihypertensive medication with circulating markers of inflammation

In univariable analyses in women, use of antihypertensive medication was associated with higher levels of hs-CRP, neopterin and KTR (all *p* < 0.05) (data not shown). In adjusted analysis, antihypertensive medication was only associated with higher levels of neopterin in women (*p* < 0.05) (data not shown). In univariable analyses in men, antihypertensive medication was associated with higher levels of hs-CRP (*p* < 0.05) (data not shown). After multivariable adjustments, antihypertensive medication was not associated with circulating markers of inflammation in men (all *p* > 0.05) (data not shown). We did not have power to look at individual antihypertensive medications.

### Subgroup analyses among participants with an increase in BP during follow-up

Over 6 years follow-up, 54% of women and 44% of men had an increase >0 mmHg in systolic BP. Our main results remained significant among participants with an increase in systolic BP during follow-up; higher levels of hs-CPR remained significantly associated with higher systolic and diastolic BP and increases in systolic and diastolic BP in women (all *p* < 0.05) and was still not associated with BP or increases in systolic or diastolic BP in men (all *p* > 0.05) (data not shown). Likewise, higher levels of neopterin remained associated with higher levels of diastolic BP and increase in diastolic BP in women (both *p* < 0.05) and was still not associated with diastolic BP or increase in diastolic BP in men (both *p* > 0.05). In contrast, in this subgroup, higher levels of PAr were associated with higher systolic and diastolic BP and increases in systolic and diastolic BP in women (all *p* < 0.05) but was not associated with BP or increase in BP among men (all *p* > 0.05) (data not shown).

Only 30% of women and 40% of men had an increase >0 mmHg in diastolic BP during follow-up. No significant associations between the circulating markers of inflammation and diastolic BP or increase in diastolic BP was found in this subset in either sex (all *p* > 0.05) (data not shown).

### Sensitivity analysis

In sensitivity analysis excluding participants with previous myocardial infarction or stroke and participants taking antihypertensive medication, the associations between hs-CRP and neopterin with BP, BP change and new-onset hypertension did not change significantly. The sex-neopterin interaction in the multivariable model for diastolic BP was attenuated *p* = 0.07, while there was a significant sex-hs-CPR interaction in the multivariable model on change in diastolic BP.

## Discussion

The present study adds novel information on sex-specific associations between prognostically validated markers of inflammation and BP in early midlife. Among women, higher hs-CRP levels were independently associated with higher BP, larger 6-year BP increase and new-onset hypertension in early midlife. Likewise, higher neopterin, a marker of T cell activation, was associated with both higher diastolic BP and higher 6-year increase in diastolic BP in women. In contrast, a higher PAr index was associated with higher diastolic BP in women and with both higher systolic and diastolic BP in men.

The role of inflammation in BP development has been reported in experimental studies [5, 22]. Common hypertensive stimuli like angiotensin II and high dietary salt intake activate pro-inflammatory T cells which infiltrate the arterial wall and promote peripheral arterial dysfunction, resulting in increased vascular resistance which contributes to BP elevation [22, 23]. However, there is a paucity of clinical studies of this topic, in particular of studies using a sex-specific approach. Although healthy young women have lower average systolic and diastolic BP than their male counterparts [24], women have a steeper increase in BP than men with aging, starting already in the third decade [2]. In the current study, women experienced an increase in systolic BP, whereas men had decreasing levels of both systolic and diastolic BP over 6 years in their forties. Our study expands earlier findings by demonstrating that circulating markers of inflammation are associated with BP and BP change in early midlife in a sex-specific manner. Taken together these results suggests a sex specific interaction between inflammation, vascular injury, and BP increase.

Chronic low grade cardiovascular inflammation is a hallmark of obesity [25]. Obesity is a common co-morbidity in hypertension, particularly in women [26]. It is therefore of special interest that the associations of higher hs-CRP with BP-increase and new onset hypertension in early midlife were independent of BMI and only found in women in the present study. These results add to knowledge from previous studies [27, 28]. In the Women’s Health Study, higher levels of CRP was associated with incident hypertension in 20,525 women aged 45 years or older [27]. However, in the Physician’s Health Study performed in men only, CRP was not associated with a higher risk of hypertension among individuals age 40–84 years [28]. Taken together these findings are in line with results from the present study, where the association between hs-CRP and systolic and diastolic BP in men was explained by higher BMI. In contrast, the association between hs-CRP and systolic and diastolic BP was independent of BMI in women.

Higher plasma neopterin was associated with higher diastolic BP, and increase in diastolic BP in women only, suggesting that T-cell-mediated inflammation may be involved in diastolic BP increase in middle-aged women. Increased level of plasma neopterin has been associated with vascular dysfunction and atherosclerosis [29]. In a small study by Zhang et al. in 24 middle-aged subjects with hypertension, higher neopterin was associated with endothelial dysfunction and higher arterial stiffness [29]. Furthermore, in 121 patients with diabetes type 2, higher circulating level of neopterin was associated with higher prevalence of carotid plaques [30]. In the Hordaland Health study, we recently demonstrated a strong association between stage 1 diastolic hypertension in early midlife and risk of acute coronary syndromes during 16 years follow-up in women [3]. In line with this, in the UK biobank study, isolated diastolic hypertension was associated with increased risk of myocardial infarction and CVD death in women, but not in men [31]. Taken together, these results indicate that vascular inflammation may contribute to increased risk for acute coronary events through diastolic BP elevation in young and middle aged women [3].

Systemic inflammation leads to altered metabolism of vitamin B6 [10]. The newly developed PAr index reflects impaired vitamin B6 metabolism and is both a marker of acute phase reaction and cellular inflammation [10]. This marker has been associated with increased risk of stroke and all-cause mortality in community based cohorts [11, 14, 32]. The present study adds to this by demonstrating that among subjects experiencing an increase in systolic BP during follow-up, higher levels of PAr were associated with higher systolic and diastolic BP in women. In the total study population, the PAr index was positively associated with systolic and diastolic BP in men, and with diastolic BP in women.

### Limitations and strengths

Our study has some important limitations. Sex hormones influence BP development and regulation through a number of mechanisms. In midlife, women experience a transition in female sex hormones. Sex hormones were not measured in our study cohort. However, the average age was 42 years at baseline, 10 years less than the average menopausal age in Norwegian women. Furthermore, inflammatory markers were only measured at the end of the 6 years follow-up. Thus, the prospective associations between circulating levels of inflammatory markers and BP development could not be tested. The strengths of the study include the wide selection of inflammatory markers, the relatively large and unselected community-based study population and the sex-specific analysis approach.

## Conclusion

In the Hordaland Health study, higher levels of circulating markers of inflammation, including hs-CRP, neopterin and the PAr index, were associated with higher BP, BP change and new onset hypertension in a sex-specific manner. After adjustment for BMI, higher hs-CRP was associated with higher BP and new onset hypertension only in women. Neopterin was associated with diastolic BP in women, while PAr was associated with BP in women and men. Our results suggest that there is a sex-specific interaction between vascular inflammation and BP in midlife.

## Summary table

### What is known about the topic

- Age-associated blood pressure (BP) development differs between women and men. Among young adults, women have lower BP than men. Then, starting from the 3rd decade, women have a steeper increase in BP than men.
- BP is a stronger risk factor for coronary heart disease in women than men, and this risk initiates at a lower BP level in women than men.
- Inflammation is associated with higher risk of hypertension.

### What this study adds

- Among 48-year-old participants in the Hordaland Health study, higher levels of circulating markers of inflammation were associated with higher BP and new-onset hypertension in a sex-specific manner.
- Higher levels of plasma hs-CRP and neopterin were associated with new onset hypertension and higher BP only in women. Our results suggest a sex-specific interaction between BP and vascular inflammation in midlife.

## Data Availability

The dataset used in this study contains potentially sensitive information. The Regional Committee for Medical and Health Research Ethics does not allow for public deposition of the data. Application for access to the data can be done on the HUSK website: https://husk-en.w.uib.no/how-to-apply-for-data-access/.
